# Research on denoising method of chinese ancient character image based on chinese character writing standard model

**DOI:** 10.1038/s41598-022-24388-y

**Published:** 2022-11-17

**Authors:** Miao Yalin, Liang Li, Ji Yichun, Li Guodong

**Affiliations:** School of Printing, Packaging and Digital Media, Xi’an University of Technology, Xi’an, 710048 China

**Keywords:** Neuroscience, Engineering

## Abstract

Ancient documents are historical evidence of cultural inheritance, and the damage brought by natural and human factors to ancient documents is inevitable, resulting in the collected images of ancient Chinese characters containing a large amount of noise, which seriously affects the accuracy of subsequent image recognition and thus creates a great obstacle to the digitization of ancient documents. To address the complexity of ancient text structure, this paper proposes a Chinese ancient text image denoising method based on the Chinese character writing standard model. The method firstly adds four additional local branches based on the global branching, and uses the supplementary character detail information to weaken the phenomenon of strokes adhering to noise due to the lack of local details; secondly, it introduces the simulation noise of ancient documents to simulate the real ancient character image morphology, which can be used for the adversarial training of this method. In the training process, the minimum absolute value deviation, smoothing loss, structural consistency loss and the refined loss function formed by the adversarial loss are used to iteratively optimize the parameters. Finally, experiments prove that the model in this paper can increase the peak signal-to-noise ratio (PSNR) and structural similarity (SSIM) of the image by at least 23.8% and 11.4%, and the user evaluation index (UV) has also reached more than 80%.

## Introduction

This subsection focuses on the necessity of targeting ancient texts for denoising.The ancient documents are the historical evidence of cultural heritage. Due to natural and human factors, different carriers of ancient documents have been damaged to different degrees, and the collected images contain a lot of noise. The progress and development of science and technology have brought convenience to society. With the power of technology, the noise in ancient Chinese character images can be effectively removed to help restore the original appearance of ancient Chinese texts, and at the same time, the integrity of the ancient character structure is preserved and sufficient preparation is made for the subsequent image processing work. In this paper, we will mainly focus on the problem of noise removal from ancient Chinese character images.

In the traditional sense, researchers have proposed a filter for image denoising. The premise is to know the specific types of noise in the image. The common types of noise are Gaussian noise, Poisson noise, speckle noise and salt and pepper noise. In 2016, amitha et al^1^ proposed that adaptive Gaussian filter and median filter should be combined to apply to the inscription images containing various noise types. Although important phase information is retained,the effect of denoising was seriously affected by the large damage of some inscription images, which resulted in missing text strokes in the final denoising results. In the same year, Shi et al^2^ analyzed the structure and characteristics of different types of inscription images and selected suitable filters to process them in order to highlight the structural part of Chinese characters, but the method is not universally applicable.

Deep learning has been widely used in image denoising due to its powerful feature learning capability^3^.In 2017, Jiao et al^4^ proposed to apply Deep Convolutional Neural Network (DCNN) to image denoising for feature extraction and learning. Experimental results showed that the network can effectively denoise images in high noise environments and has a greater advantage over traditional filter-based methods.In 2014, Dr. Goodfellow et al^5^ introduced Generative Adversarial Network (GAN) into the field of deep learning, inspired by the idea of two-player zero-sum game in which the sum of the benefits of both players is constant and the loss of one player is equal to the gain of the other, so that it GAN^6^ uses adversarial learning, where the discriminator learns the difference between the generative distribution and the true distribution, and then drives the generator to reduce the difference. In contrast to other generative networks that do not impose explicit restrictions on the distribution of the data, thus avoiding the need to manually design the network distribution.In 2020, Zhang et al^7^ improved the generative adversarial network by combining convolutional neural networks to perform page-level denoising of calligraphy works, minimizing the artifacts introduced during denoising while preserving good Chinese character structure.

**Figure 1:**
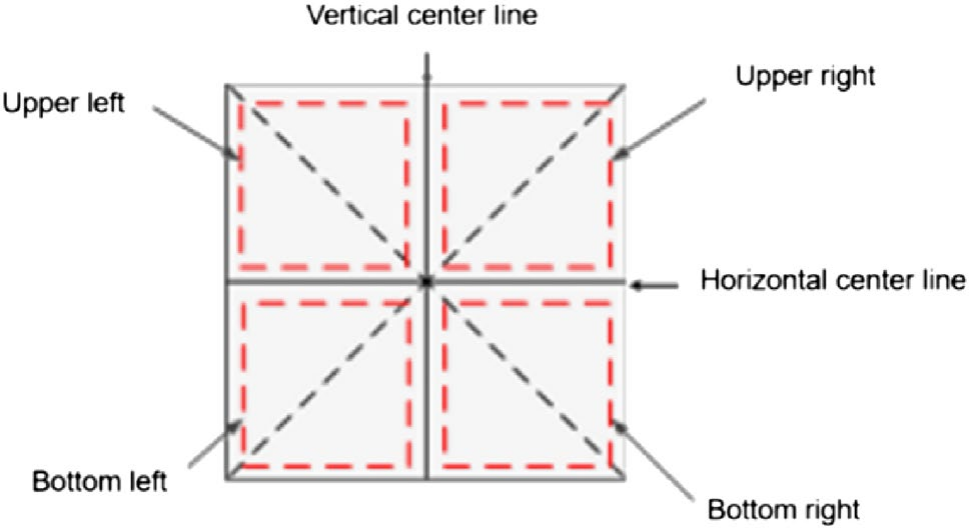
Tianzig.

Since the beginning of our exposure to Chinese characters, we have also been exposed to the "Tianzig" model shown in Fig. 1, which enables us to get a better sense of the radicals and overall structure of each character, and to standardize the steps of writing Chinese characters.In this paper, we propose a method for denoising images of ancient Chinese characters based on the model of Chinese character writing standard, drawing on "Tianzig".The main contributions are: (1) the noise in Chinese ancient character images is naturally formed, and no satisfactory original images have been preserved historically. To address the problem of the lack of datasets of real images of Chinese ancient character, this paper introduces an ancient document simulation noise to simulate the real ancient character image morphology and form a new paired dataset with the original target images for adversarial training of the network model; (2) For the the complexity of ancient character structure, combined with the Chinese character writing specification model (Tianzig), this paper proposes a Chinese ancient character denoising model based on the Chinese character writing specification model. The model firstly introduces a channel attention mechanism to focus on the key regions of the image during feature extraction, secondly generates global and local Chinese character structure images after denoising, and finally fuses the two by a refinement processor to complete the complement of Chinese character detail information and weaken the phenomenon of strokes sticking to the noise due to the lack of local details.

## Related work

This subsection introduces the related GAN network to lead to the basic network CGAN^8^ of this paper.The main advantage of GAN is that it goes beyond the traditional neural network classification and feature extraction to generate new data according to the characteristics of real data. As the two networks progress in the adversarial process and continue the adversarial process afterwards, the data obtained by the generative network becomes more and more perfect and approximates the real data, so that the desired data (pictures, sequences, videos, etc.) can be generated. The generator generates synthetic data from a given noise (generally a uniform or normal distribution). It tries to produce data closer to the real one. The most serious problem of neural networks is that they do not have the ability to explain their reasoning process and the basis of reasoning; they cannot ask the necessary queries to the user, and they cannot work when the data is not sufficient; they turn all the characteristics of the problem into numbers and all the reasoning into numerical calculations, which inevitably results in the loss of information; the theory and learning algorithms need to be further improved and enhanced. So here we have chosen to generate an adversarial network and thus used the convolutional layer of the generator.

### Generating adversarial networks

**Figure 2:**
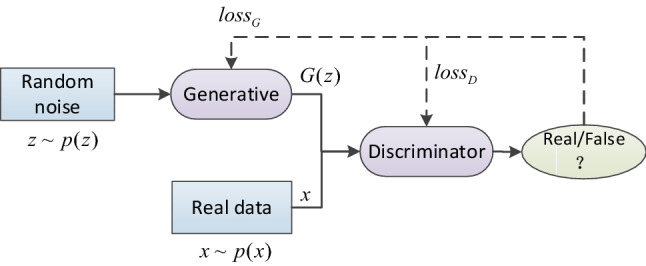
Generative Adversarial Network.

The network structure of the generative adversarial network is shown in Fig. 2. Two game players are generator (G) and discriminative (D). Both of them make the data distribution of generator as close to the real data distribution as possible through continuous "game", thus "cheating" discriminator. Generator G is mainly used to capture the distribution of real data samples, and to obtain the generated image by receiving a random noise sampled from prior distribution. Discriminator D is used to distinguish the input data and output the probability that the input image is a real image. If the result is 1, it means that it is a real image, otherwise it means a faked image.

In the training process, the purpose of judging network D is to judge true and false as much as possible. When a true image ${\text{x}}$ is given, network D is expected to give a high score, while for the image $G({\text{z}})$ generated by generator G, discriminator D is expected to give a low score as far as possible, that is, the calculated $D(G({\text{z}}))$ score is as small as possible. Therefore, the generation of the objective function of the adversarial network is the maximization and minimization process of the objective function, as shown in Eq. (1) :

1 $$\begin{aligned} \mathop {\min }\limits_{G} \mathop {\max }\limits_{D} V(D,G) & = E_{{x\sim P_{data} (x)}} [\log D(x)] \\ & \quad + E_{{z\sim P_{z} (z)}} [\log (1 - D(G(z))) \\ \end{aligned}$$

where $P_{{{\text{data}}}}$ represents the distribution of real data; $P_{z}$ is noise distribution; $G(z)$ represents the false data generated by the generator model based on random noise ${\text{z}}$, which is close to the real data;$D(x)$ represents the probability that the real data judged by the discriminant model is true; $D(G(z))$ represents the probability that the model judges the fake data to be true.

### Conditionally generated adversarial network

**Figure 3:**
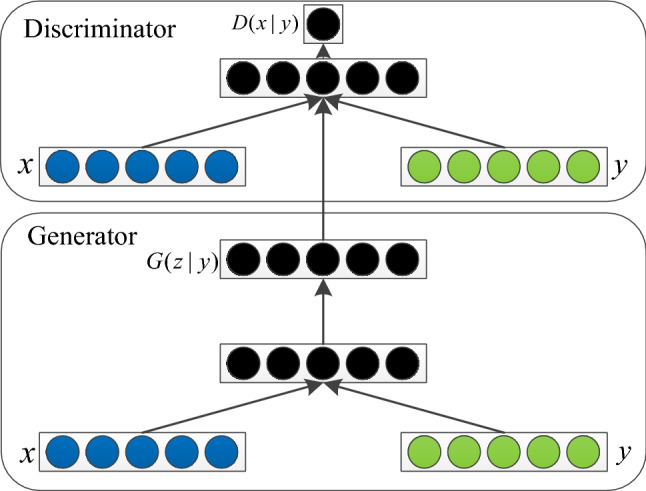
Conditional Generative Adversarial Nets.

GAN becomes uncontrollable in the face of larger images and complex data because the intermediate simulation is too free, so Mirza et al^8^ proposed Conditional Generative Adversarial Nets (CGAN) in 2014, which introduces in the generator network and discriminator network Conditional Variable (CV) was introduced into the generator network and discriminator network, so that the GAN is trained using images with corresponding labels to make the generated data and the real data match better, and the network model is shown in Fig. 3.

**Figure 4:**
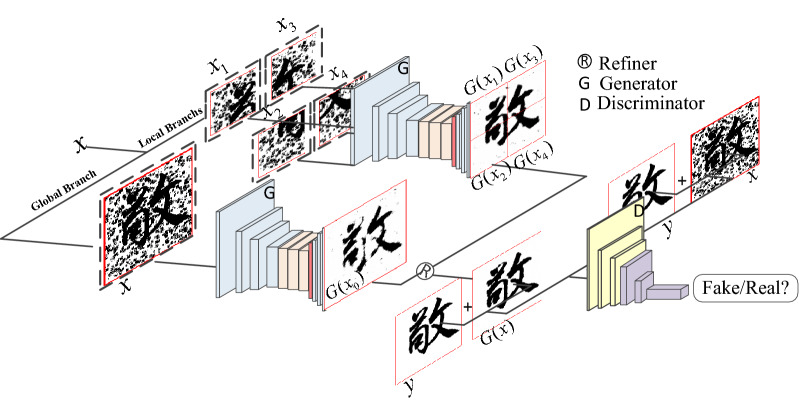
The overall structure of the Chinese character writing standard model.

As can be seen from Fig. 2, 3 and 4, the network of CGAN does not change compared with the original GAN network, only the input data of generator G and discriminator D are changed. Therefore, CGAN can be embedded into other GAN networks as a general strategy. The loss function obtained is shown in Eq. (2) :

2 $$\begin{aligned} \mathop {\min }\limits_{G} \mathop {\max }\limits_{D} V(D,G) & = E_{{x \sim P_{data} (x)}} [\log D(x|y)] \\ {\kern 1pt} & \quad + E_{{z \sim P_{z} (z)}} [\log (1 - D(G(z|y)))] \\ \end{aligned}$$

In general, GAN can be applied in many fields because of its flexible learning ability.However, for GAN, it is often difficult to control the training and cannot generate the desired results. Conditional generative adversarial network CGAN in order to change this situation, so it is necessary to restrict the output and input of generative adversarial network, input the conditional label of control content into the generator and discriminator, or add other conditional information to restrict the input to the generator. Therefore, the generator network of the proposed denoising model in this paper will borrow the idea of CGAN and use a symmetric convolutional neural network structure to apply to thedenoising of ancient Chinese character images.

## Chinese ancient character image denoising method based on chinese character writing standard model

This subsection focuses on the construction of the correlation model, the fusion of local and global branches, and the loss function.

### Model framework

In order to obtain high quality image denoising effect and reduce the noise residue near the stroke structure of ancient characters. In this paper, from the perspective of preserving the integrity of ancient script structure, the Chinese ancient character images are denoised by the Chinese character writing standard model.and uses the obtained local information to supplement the global information in detail. The overall framework is shown in Fig. 4, and the specific implementation process is as follows:

1. The input of the model adds four additional local branches to the global branches, and the overall image of the Chinese ancient character is input to generator G to capture the most significant global noise and remove it, while the Chinese ancient character image is cropped in equal parts according to the midpoint of the width and height, and then input to generator G in a progressive manner to remove the noise near the local Chinese character stroke structure, where each generator G shares the same architecture, focusing on the focal region by adding a channel attention mechanism.
2. The generated image ($G(x_{1} ),G(x_{2} ),G(x_{3} ),G(x_{4} )$)of the four local branches is stitched into an image according to their respective positions, and then the stitched image is merged with the generated image ($G(x_{0} )$)of the global branch to generate the final denoising image $G(x)$. Finally, {$G(x),y$},{$x,y$} are input to the discriminator D to determine the authenticity of the data composed of the source image and the target image respectively.

### Generator network

**Figure 5:**
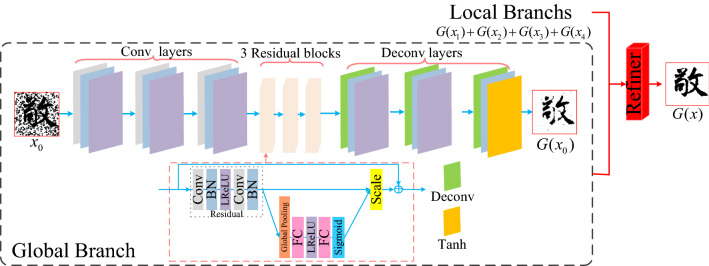
Generator network for Chinese character writing standard model.

The generator network and discriminant network of CGAN can learn the representation hierarchy of objects from part to scene, add constraint conditions to the model with the added label information, guide the data generation process, and apply the learned features to new tasks to prove their applicability as general image representations. The generator network in this chapter draws on the idea of CGAN and adopts the symmetric convolutional neural network as shown in Fig. 5 to learn end-to-end mapping directly from the input image and its corresponding real image, so as to improve the visual effect of the denoising image.

The overall structure of the generator network is divided into three main parts: the downsampling layer, the attention residual module, and the upsampling layer. In the downsampling part of the network, no pooling layer is used, and three convolutional layers are used to extract shallow features of the image. Each convolutional layer is followed by a batch normalization layer BN and an activation function layer LReLU to increase the nonlinearity of the network and thus accelerate the convergence. The core migration layer is constructed using residual blocks that introduce a channel focus mechanism to help the network focus on key regions and enhance the information transfer between layers so as to extract deeper hidden information.The final upsampling layer uses deconvolution, the first two groups both use the LReLU activation function, and the final output layer uses the Tanh activation function. Among them, the local branching can better preserve the local Chinese character structure details, but ignore the complete structure of the whole chinese ancient character. In this paper, we introduce the refinement processor (Refiner) proposed in the literature^9^, which is applied to face facial expression conversion, and firstly, the outputs of the four local branches are pixel-filled and stitched into one image according to their respective positions in the source Chinese ancient character image. Then, the stitched image and the output of the global branch are provided to the convolutional layer, which refines and fuses the two so that the local information it collects complements the global information in detail, and the final generated image is obtained after processing.

**Figure 6:**
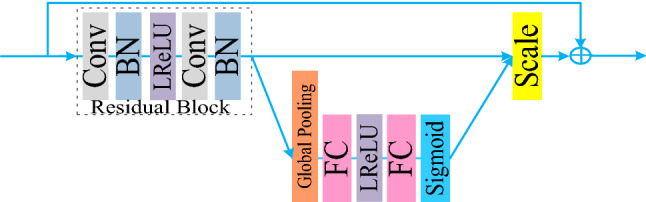
Residual Block of Fusion Channel Attention.

In convolutional neural network, the convolutional kernel is essentially the aggregation of spatial information and characteristic dimension information, which can help the network capture the global and local features of images. At present, most convolutional neural networks improve their performance through spatial dimension. This article uses the re-calibration approach, on the basis of the residual block introduced features channel attention module, make its focus on characteristics of the channel, the interdependent relationship between explicit modeling, and realign the leading feature of the adaptive response, learning the weight of each channel, according to the weight to the judgment of the next, as shown in Fig. 6.

### Discriminator network

**Figure 7:**
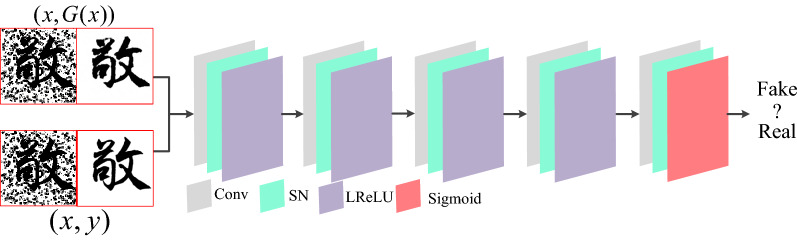
Discriminator network for Chinese character writing standard model.

The purpose of image denoising is to make the result of denoising comparable to real images in terms of visual perception and image quality. Therefore, this paper introduces a self-learning discriminator network, and gradually strengthens the ability to distinguish the authenticity of images through the iterative optimization of parameters in the network.The network structure of the discriminator is shown in Fig. 7. In the whole discriminator network, the pooling layer is replaced by a convolutional layer with step size to avoid the loss of data information caused by the pooling layer and to enhance the description of the detailed features of Chinese ancient characters. Four convolutional layers with Switchable Normalization (SN) and Leaky ReLU activation functions are used to compute and learn the features, and the last layer is compressed using the Sigmoid function to obtain a regularized probability score between [0,1], which alleviates the gradient disappearance phenomenon and increases the model's stability.

### Refinement processor

**Figure 8:**
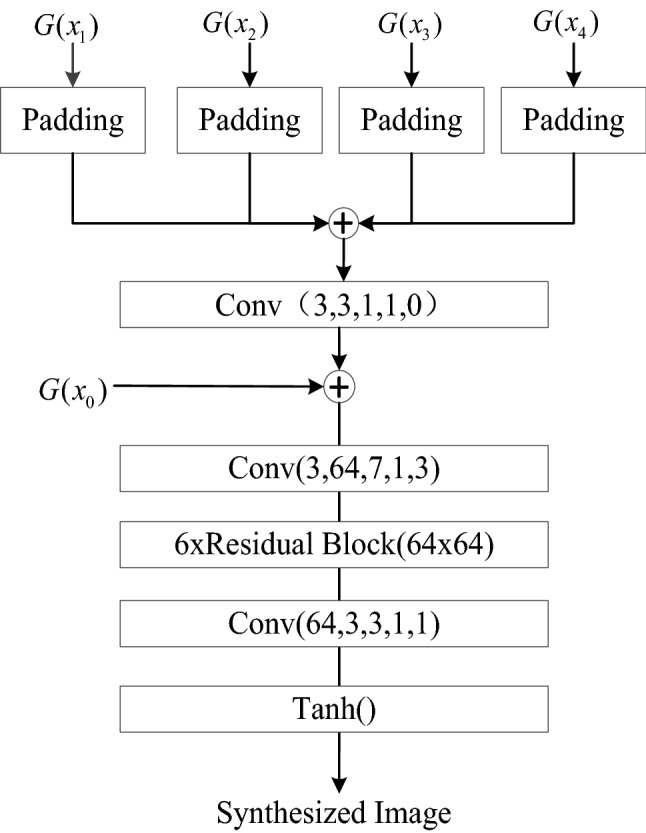
Refiner.

Local branches can better preserve local character structure details, but ignore the complete structure of the whole character. In this paper, we introduce a refinement processor (Refiner) proposed in the literature^9^ for application to face facial expression transformation, where the outputs of four local branches are first pixel-filled and stitched into one image according to their respective positions in the source character image. Then, the stitched image and the output of the global branch are provided to the convolutional layer to refine and fuse the two, so that the local information it collects details and complements the global information, and the final generated image is obtained after processing, and the overall structure of Refiner is shown in Fig. 8.

### Loss function

The loss function is an important indicator for the smooth training of the discriminator supervising generator. Traditional loss functions often use cross entropy as a pixel-level loss function, and update the generator network by obtaining the minimum value of the Mean Square Error(MSE). Although the image loss generated by MSE optimization has a high peak signal-to-noise ratio, the texture is often too smooth due to the lack of high-frequency information. In order to enable the network to generate clearer images of ancient chinese characters, this paper uses the hierarchical structure adversarial loss as the discriminative loss function. The minimum absolute error, structural consistency loss and smoothing loss are combined with appropriate weights to form a new refined loss function as the generator loss function. The definition of the refined loss function formed is shown in formula (3):

3 $$\begin{aligned} L & = L(D) + L(G) \\ &= L_{adv} + \lambda_{1} L_{1} + \lambda_{S} L_{S} + \lambda_{SSIM} L_{SSIM} \\ \end{aligned}$$

Where $L_{adv} ,L_{{1}} ,L_{S} ,L_{SSIM}$ respectively represent the adversarial loss, minimum absolute error, smoothing loss, and structural consistency loss; $\lambda_{1} ,\lambda_{{\text{S}}} ,\lambda_{SSIM}$ respectively represent the minimum absolute error weight, smoothing loss weight, and structural consistency loss weight.

The discriminator is used to discriminate whether the generated image is a real image. The adversarial loss function in this paper adopts a hierarchical discriminator, draws on the improved WGAN-GP loss function of the WGAN network^10^, and proposes to use a gradient penalty mechanism instead of weight clipping. The constraint function is gradient penalized by the differentiable function 1-Lipschitz, and the limitation of Lipschitz can be reflected by setting additional loss items. The input of the discriminator in this paper includes the source image ${\text{x}}$ and target image ${\text{y}}$ of ancient Chinese characters. The target image is used as label information ${\text{y}}$ to guide the sample generation of the generator. Its adversarial loss function is shown in Eq. (4):

4 $$L(D) = L_{{{\text{adv}}}} = \mathop E\limits_{{x\sim P_{g} }} [D(x,G(x))] - \mathop E\limits_{{x\sim P_{r} }} [D(x,y)] + \lambda \mathop E\limits_{{x\sim P_{x} }} [(||\nabla \tilde{x}D(\tilde{x},y)||_{2} - 1)^{2} ]$$

where, $\lambda$ is set to 10,$\tilde{x}$ and $x_{g}$ the random difference value sampling on the and connection line,$x_{r} \sim P_{r} ,x_{g} \sim P_{g} ,\varepsilon \sim Uniform\;[0,1]$,is shown in formula (5):

5 $$x = \varepsilon x_{r} + (1 - \varepsilon )x_{g}$$

WGAN-GP GAN further stabilize the training process to ensure the quality of the resulting image. The loss function can be used to visually show the quality and convergence of the network training. The smaller the value of the discriminator’s objective function, the more stable the network training and the better the generator optimization.

## Data set

This subsection focuses on creating the dataset for training and testing the model. In this paper,the dataset is obtained by self-constructing high-quality ancient character images in existing image database then image processing is performed and ancient document simulation noise is added according to the noise model, and the generated images containing noise and the target images form a paired dataset for the training of the network model.

### Ancient documents simulation noise

**Figure 9:**
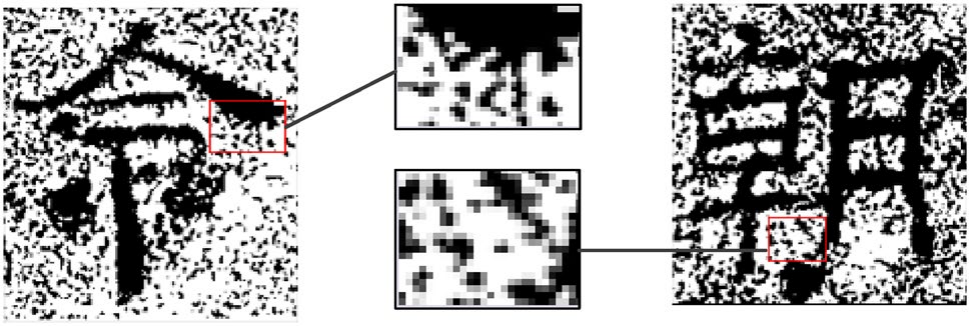
Real Chinese Ancient Characters Image.

In the process of model training, it needs to include the data pair of {source image, target image}, and the noise in the image of Chinese ancient characters in ancient documents is naturally formed, and no satisfactory original images has been preserved in history. This paper introduces ancient documents simulation noise^7^ to simulate the noise naturally formed in Chinese ancient characters in ancient documents as shown in Fig. 9. As can be seen from the figure, the shape of the real characters of the image noise block most of them are different, to closely mimic thereof herein by dots. For the original image, noise is randomly generated in a uniformly distributed manner, and then the final output noisy image and the original image are formed into a paired data set of {source image, target image} for network training.

**Figure 10:**
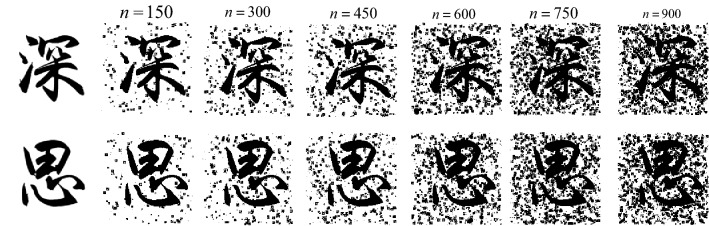
Simulation noise of different intensity of ancient document simulation.

The specific method of adding noise is shown in formula (6):

6 $$\left\{ {\begin{array}{*{20}c} {R_{0} \sim U[1,3]} \\ {(x_{0} ,y_{0} ) \in \{ \left. {(x,y)} \right|x \sim U[0,c],y \sim U[0,r]\}^{n} } \\ {n = 150,300,450,600,750,900} \\ \end{array} } \right.$$

where $(x,y)$ represents the coordinates of any pixel in the image, $r,c$ represents the number of rows and columns in the image. In the image area, Draw a circle with a radius of $R_{0}$ with $(x_{0} ,y_{0} )$ as the center. Each pixel in the circle has a probability of $P$ may be a noise point. Here, set $P$ to 0.8. $R_{0},x,y$ obeys a uniform random distribution. In addition, in order to increase the diversity of noise, the noise in the rectangular area is added on the basis of the noise in the circular area. The specific expression is shown in (7):

7 $$\left\{ {\begin{array}{*{20}c} {R_{1} \sim U[1,6]} \\ {(x_{1},y_{1} ) \in \{ \left. {\left( {x,y} \right)} \right|x \sim U[0,c],y \sim U[0,r]\}^{n} } \\ {n = 150,300,450,600,750,900} \\ \end{array} } \right.$$

where $R_{1}$ represents the boundary length of the rectangle as the center $(x_{1},y_{1} )$; $P$ represents the probability that each pixel in the rectangle may be a noise point, set to 0.8; $R_{1},x,y$ obeys a uniform random distribution. As shown in Fig. 10, the result of adding different intensities of noise to the original noise-free image.

### Experimental data set

**Figure 11:**
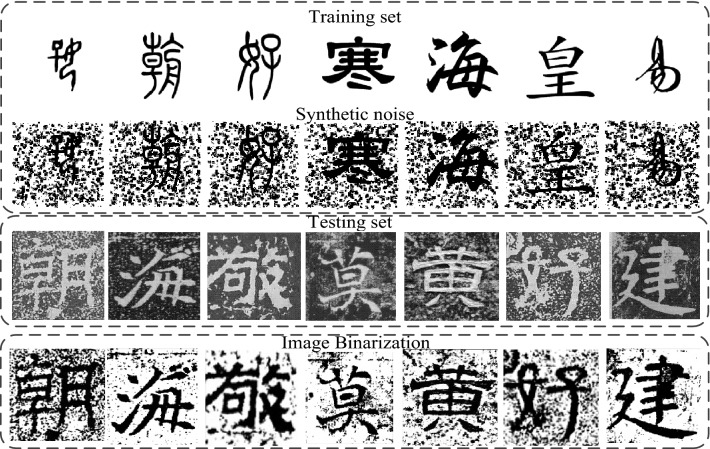
Data Set.

This paper uses data generation technology to build a data set for the training of the image denoising model of Chinese ancient characters. The overall data set is shown in Fig. 11.

The data set covers seven fonts (oracle bone inscriptions, bronze inscriptions, seal script, official script, running script, regular script, and cursive script) of the evolution of Chinese characters^11^. Each font contains 100 Chinese characters. The first line is the original data set. Introduce ancient documents simulation noise for each Chinese character image in the data set (the noise intensity is 600 in the figure) to simulate the real Chinese ancient character image of ancient documents, as shown in the second row, form a total of 700 pairs (1400) of training data sets with the original data set. The real ancient character images are shown in the third row, which are binarized and combined with some of the datasets with added ancient documents simulation noise in a 1:1 ratio to form the final test set with a total number of 100.

### Model training

**Figure 12:**
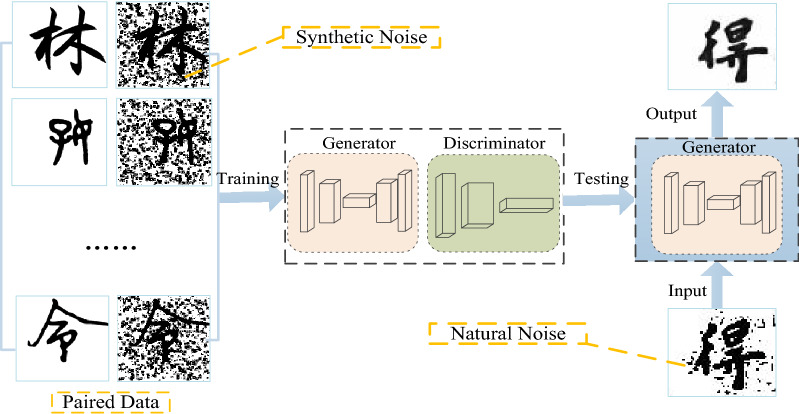
Training of Network Mode.

The training is to predict the denoised image from the noisy image, and the discriminator is to distinguish the match between the denoised image and the real image by outputting a probability value. When the generator generated image de-noising enough to deceive the discriminator, this indicates the end of training. Finally, the trained network is formally applied to the denoising processing of real Chinese ancient character images, as shown in Fig. 12 for the training process of the model in this paper.

As can be seen from the figure, first initialize the parameters of the two networks of generator G and discriminator D. Secondly, n samples are generated for the samples in the training set using the defined ancient literature simulation noise to form a paired data set; finally, the weights of the loss function are set to $\lambda_{1} = 10,\lambda_{S} = 1,\lambda_{SSIM} = 1$, the parameters are updated using the Adam optimization algorithm, and the first-order exponential decay rate $\beta_{1}$ and the second-order exponential decay rate $\beta_{2}$ of the optimizer are set to 0.5 and 0.9, respectively. The learning rate is set to 0.0001, the number of batch samples is set to m = 1, the number of iterations is set to T = 200, and the entire training sample set is traversed according to the number of iterations.

## Experimental results and discussion

This subsection focuses on the comparative experimental validation of different methods, different noise types, and different loss functions.

### Experimental environment

The model in this article is based on the open source deep learning library TensorFlow. Due to the large number of matrix operations in convolution operations, this article uses Graphics Processing Unit (GPU) instead of Central Processing Unit (CPU) to improve computing efficiency. The specific experimental environment parameters are shown in Table 1:

**Table 1:** Experimental environment parameters.

Name	Parameter	Name	Parameter
Cpu	Intel Core i7-7700	Running memory	32 GB
GPU graphics card	Nvidia GeForce GTX 1080	disk space	4 TB
Python version	3.7.7	CUDA version	10.0
Tensorflow version	1.14.0	Threads	Eight threads
Operating System	Ubuntu 20.04 LTS	CuDNN version	7.4

### Evaluation indicators

The evaluation indicator in this paper uses a combination of subjective and objective evaluation indicators to measure the effectiveness of the denoising model, where the subjective visual evaluation indicator uses user research evaluation (UV), and the objective evaluation indicator uses peak signal-to-noise ratio (PSNR) and structural similarity (SSIM).

1. User survey and evaluation (UV)

User survey evaluation (User Version, UV) is a quantitative representation of visual evaluation, which intuitively reflects the user's subjective evaluation of the quality of the generated denoising image. This indicator evaluates and counts the denoising results generated by a certain number of users and different denoising models, and selects Chinese ancient character images with good denoising effects and visual aesthetics from the set number of generated results. Its quantified expression is shown in formula (8):

8 $$UV = \frac{n}{N \times M}$$

In the formula:$n$ indicates the number of images rated as the best effect;$N$ indicates that $N$ user is selected for evaluation ($N = 10$);$M$ indicates that the test results of each model are randomly selected$M = 20$.

(2) Peak Signal-to-noise Ration

Peak Signal to Noise Ratio (PSNR) is used to measure the difference between the corresponding pixels of the reconstructed image and the real image, and is one of the commonly used objective evaluation indicators for images.In ancient character image denoising, the larger the PSNR value, the more consistent the denoised image is with the target image, indicating that the denoising effect is better and the distortion effect is smaller. The measurement unit of PSNR is dB, and its calculation process is shown in formula (9):

9 $$PSNR{ = }10\log \left( {\frac{{L^{2} }}{MSE}} \right)$$

In formula (9), MSE is the mean square error (Mean-Square Error), which is used to calculate the mean value of the square of the difference between the pixels at the same position in the real image and the denoised image. The specific calculation formula (10) is as shows:

10 $$MSE{ = }\frac{{\sum\limits_{x = 1}^{M} {\sum\limits_{y = 1}^{N} {[X(x,y) - \hat{X}(x,y)]^{2} } } }}{MN}$$

where $X$ represents the reference image; $\hat{X}$ represents the image to be evaluated.

(3) Structural similarity

Structural Similarity Index (SSIM) is one of the objective evaluation indicators used in this paper, which is mainly used to measure the degree of similarity between two images. It usually ranges from 0 to 1, and the result is 1 when the two images are the same, and the mathematical expression is shown in formula (11).

11 $$SSIM\left( {x,y} \right) = \frac{{\left( {2\mu_{x} \mu_{y} + c_{1} } \right)\left( {2\sigma_{xy} + c_{2} } \right)}}{{\left( {\mu_{x}^{2} + \mu_{y}^{2} + c_{1} } \right)\left( {\sigma_{x}^{2} + \sigma_{y}^{2} + c_{2} } \right)}}$$

### The experimental results

1. The influence of different intensities of noise on the denoising effect

**Figure 13:**
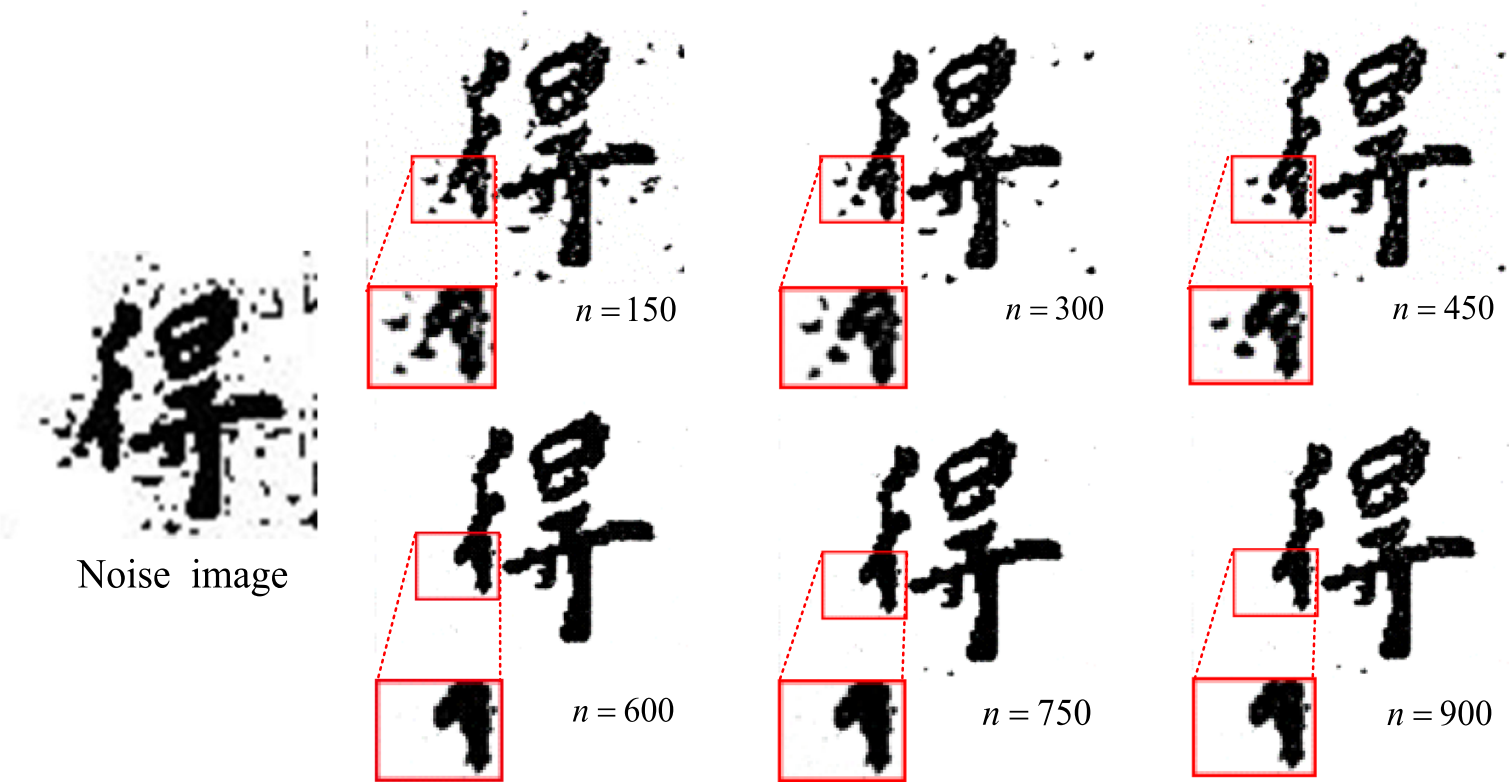
Denoising Results of Artificial Noise With Different Intensities After Model Training.

Different intensity of simulation noise will affect the result of real ancient text image denoising. By adding noise without intensity for the training of the network model, and testing through real ancient text images, the results are shown in Fig. 13. The symbol ${\text{n}}$ represents the noise intensity, and the red box represents the amplification of the details of the resulting image after denoising. With the increase of noise intensity (${\text{n}}$ = 150 ~ 900, spacing is 150), the denoising effect also changes. At the initial stage, with the increase of noise intensity (${\text{n}}$ = 150–600), the denoising effect is continuously enhanced, which can effectively reduce the stroke holes produced in the process of denoising ancient text images. After ${\text{n}}$ > 600, the denoising effect tends to be stable and even decreases with the increase of noise intensity. As can be seen from Tables 2 and 3, when ${\text{n}}$ = 600, it is comparable to SSIM value of ${\text{n}}$ = 750, but PSNR value is slightly higher than UV value of user evaluation index. Therefore, noise with intensity of 600 is determined to be added in this paper for the construction of paired dataset.

(2) The influence of different types of noise on the denoising effect

**Table 2:** Index evaluation results of different intensities of noise.

Evaluation index	$n$ = 150	$n$ = 300	$n$ = 450	$n$ = 600	$n$ = 750	$n$ = 900
PSNR(${\text{dB}}$)	16.0127	16.8549	17.8094	19.4697	19.3374	19.2137
SSIM	0.7053	0.6609	0.8283	0.8567	0.8635	0.8498
UV	46%	51%	70%	82%	78%	73%

**Table 3:** Index evaluation results of different types of noise.

Noise type	PSNR(dB)	SSIM	UV (%)
Salt and Pepper Noise	16.7842	0.7830	59
Gaussian Noise	17.6168	0.6906	66
Synthetic Noise	19.4697	0.8567	78

**Figure 14:**
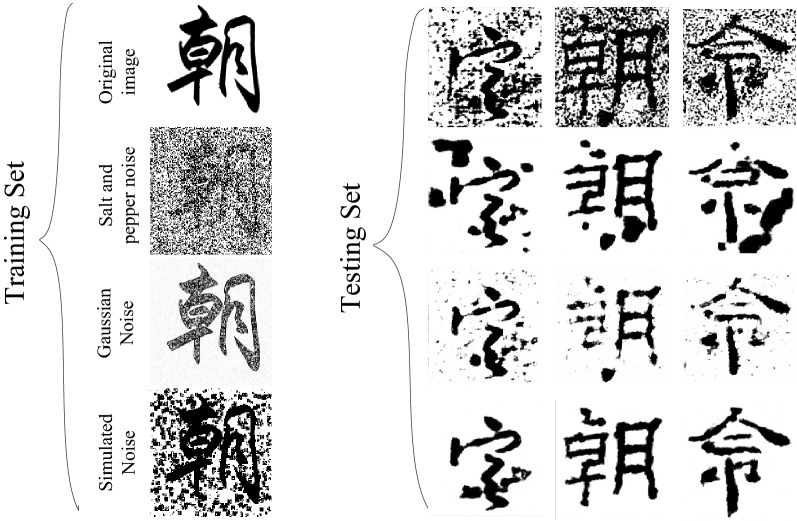
Denoising Effect of Different Noises after Model Training.

In order to verify the usability of the simulated noise of ancient documents introduced in this paper in the study of ancient character image denoising. On the basis of the previous experiment, Gaussian Noise with a variance of 0.001 and Salt and Pepper Noise with a noise value of $prob$ = 0.12 were added to the original data set for noise processing. A paired data set is formed for the training and testing of the model in this paper, and the test results are shown in Fig. 14.

As shown in the figure, the first column represents the processing results of adding different noises to the training set, and the second column and later represents the denoising results after adding different types of noises to the training network. It can be seen from the figure that the denoising effect of adding Gaussian noise makes the whole Chinese characters still have a lot of noise residues. At the same time, due to excessive denoising, there is a lack of strokes in the whole Chinese ancient character strokes, which runs counter to the idea of ensuring the integrity of the Chinese character structure in ancient books; adding salt and pepper noise for network training, although the Chinese character structure is better preserved, but it makes the result figure A blocking noise that didn't exist before was generated. It can be seen from Table 3 that adding Simulation noise for network training denoising can increase PSNR and SSIM by at least 1.8529 dB and 0.0730. The overall denoising effect is better, and the structure of Chinese characters is clearer by visual observation.

(3) Comparative experimental analysis of different models

**Figure 15:**
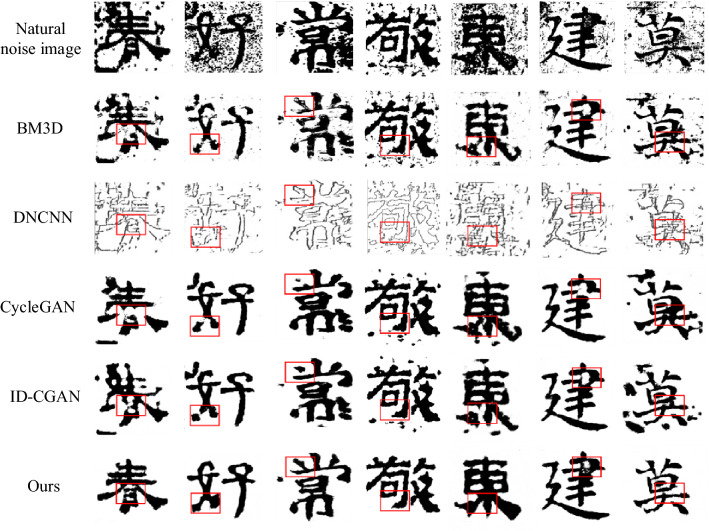
Denoising Effect of Different Methods.

The model in this paper is compared with other four image denoising models (BM3D^12^, DNCNN^13^, CycleGAN^14^, ID-CGAN^15^) through experiments. The experimental results are shown in Fig. 15.

It can be seen from Fig. 14 that the overall image after denoising by the BM3D model looks blurry. The analysis may be due to the smooth details generated in the process of joint filtering and inverse transformation resulting in the loss of information;DNCNN model can retain the outline structure of Chinese characters and noise well, but it will cause the overall loss of the internal structure of Chinese character strokes.The overall denoising effect of the CycleGAN model is significantly improved compared to the above two methods, but it removes the noise near the Chinese character structure and causes the loss of the local stroke structure; the ID-CGAN model is used for denoising, and the noise is still not completely eliminated but retained Some details of the structure of Chinese characters. The existence of image noise greatly affects visual perception and evaluation indicators. The model in this paper can effectively remove the noise that adheres to the structure of Chinese characters while retaining the complete outline and detailed structure of Chinese characters. It can be seen from Table 4 that compared with other models, the two evaluation indexes of PSNR and SSIM have increased by at least 4.2208 dB and 0.0945 for the model in this paper, and the user evaluation indexes have reached more than 80%.

(4) Experimental analysis of loss function ablation

**Table 4:** Evaluation results of metrics for different models of denoising.

Model name	PSNR	SSIM	UV (%)
BM3D	11.2175	0.5487	52
DNCNN	9.4680	0.3605	36
CycleGAN	16.8095	0.8282	69
ID-CGAN	17.7276	0.8163	72
Ours	21.9484	0.9227	86

**Figure 16:**
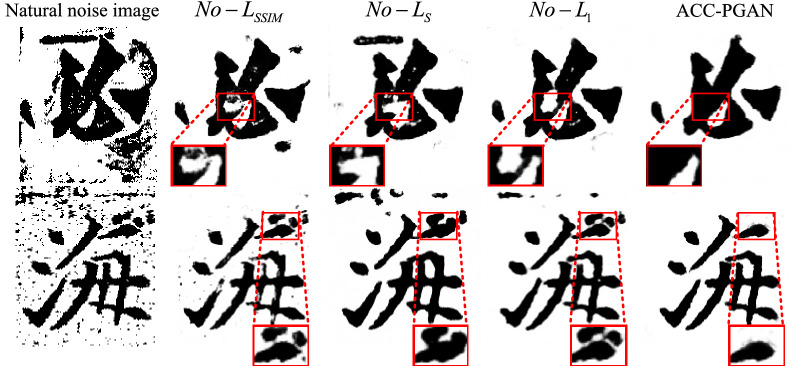
Ablation Experiment of Generator Loss Function.

In order to ensure that the generated Chinese character denoised image can have a good visual effect and quality score, the model in this paper adds the minimum absolute error, smooth loss function $L_{S}$ and structural consistency loss function $L_{SSIM}$ based on the discriminator’s anti-loss function to form a refined loss function. In this paper, the denoising effect of Chinese character image is investigated when any one of thinning loss functions is removed. The denoising results and index evaluation results are shown in Fig. 16 and Table 5.

**Table 5:** Generation of network loss function ablation experiment evaluation index.

Loss function	PSNR	SSIM	UV (%)
$No - L_{SSIM}$	16.8095	0.8282	57
$No - L_{S}$	14.9921	0.7786	49
$No - L_{1}$	17.0036	0.8362	62
Ours	21.9484	0.9227	86

The first column in Fig. 16 is the real image of Chinese ancient characters in ancient books, After that, each column represents the denoising results when using different loss functions, and the red frame represents the enlarged details of the Chinese character image after denoising. It can be seen from the above that the lack of structural consistency loss function will lead to the fracture of the stroke structure of Chinese characters; the lack of smooth loss function will cause the residual noise of the overall Chinese character structure to stick to the strokes. Compared with the denoising results of the model in this paper, It is proved that the addition of smoothing loss function can ensure good image quality and structure characteristics.; the lack of $L_{1}$ loss function can completely retain the overall outline of the Chinese character, but it is easy to cause the loss of high-frequency information; the experimental results show that the composite thinning loss function used in this article is in three indicators The evaluation results all reached the best, which can effectively eliminate noise while retaining the integrity of the structure of Chinese characters.

## Conclusion

In this paper, we propose a method to denoise ancient Chinese characters based on the Chinese writing specification model, taking into account the complexity of the structure of ancient Chinese characters, the influence of the structural integrity of Chinese characters on the recognition of Chinese characters, and the "Tianzig" and generative adversarial networks. Firstly, on top of the global branches, four additional local branches are added according to the Chinese character writing standard model, a symmetric convolutional neural network is used as the generator network, and a feature channel attention mechanism is introduced in the feature extraction layer to focus on the key regions. Secondly, adaptive normalization is used in the discriminator network to prevent the gradient from disappearing due to the sudden change of the discriminator network during the training process, thereby improving the stability of the entire model;Use the simulated ancient literature to simulate noise again, and add noise to the data set to form a new data set, which is used for the model's adversarial training. Through subjective and objective evaluation indicators, comparative experiments are carried out on different noise intensities, different noise types, different methods, and different loss functions. The noise removal effect of this model is examined from different perspectives, and it is demonstrated that this model can remove the noise adhering to the stroke structure while weakening the holes existing in the strokes after the noise removal to ensure the integrity of the Chinese character structure.Finally, experiments prove that the model in this paper can increase the peak signal-to-noise ratio (PSNR) and structural similarity (SSIM) of the image by at least 23.8% and 11.4%, and the user evaluation index (UV) has also reached more than 80%.

## Data Availability

The datasets generated and/or analysed during the current study are not publicly available as the data also forms part of an ongoing study but are available from the corresponding author on reasonable request.
